# Endoscopic mucosal resection and endoscopic submucosal dissection with an external additional working channel (EMR+ and ESD+) are equivalent to using a double-channel endoscope: a systematic evaluation in a porcine ex vivo model

**DOI:** 10.1007/s00464-023-10295-4

**Published:** 2023-08-11

**Authors:** Richard F. Knoop, Ahmad Amanzada, Golo Petzold, Volker Ellenrieder, Michael Engelhardt, Albrecht Neesse, Sebastian C. B. Bremer, Steffen Kunsch

**Affiliations:** 1 Department of Gastroenterology, Gastrointestinal Oncology and Endocrinology, University Medical Center Göttingen, Georg-August-University, Göttingen, Germany; 2 Department of Gastroenterology, Internal Medicine and Geriatrics, Rems-Murr-Hospital, Winnenden, Germany

**Keywords:** Endoscopic submucosal dissection (ESD), ESD+, Endoscopic mucosal resection (EMR), EMR+, Additional working channel (AWC), Double-channel endoscope, RESECT+, Animal model, EASIE-R model

## Abstract

**Background and aims:**

With an external additional working channel (AWC) endoscopic mucosal resection (EMR) as well as endoscopic submucosal dissection (ESD) can be extended to techniques termed “EMR+” and “ESD+.” These novel techniques are systematically compared to EMR and ESD under the use of a double-channel endoscope (DC).

**Methods:**

Our trial was conducted prospectively in a pre-clinical porcine animal model (EASIE-R simulator) with standardized gastric lesions measuring 3 or 4 cm.

**Results:**

EMR+ and EMR DC showed both good results for 3 cm lesions with no adverse events and an en bloc resection rate of 73.33% (EMR+) and 60.00% (EMR DC, *p* = 0.70). They came to their limits in 4 cm lesions with muscularis damages of 20.00% (EMR+), 13.33% (EMR DC, *p* ≥ 0.99) and decreasing en bloc resection rates of 60.00% (EMR+) and 46.67% (EMR DC, *p* = 0.72).

ESD+ and ESD DC were both reliable concerning en bloc resection rates (100% in all groups) and adverse events (0.00% in 3 cm lesions, 12.50% muscularis damages in both ESD+ and ESD DC in 4 cm lesions).

Resection time was slightly shorter in all groups with the AWC compared to DC although only reaching significance in 3 cm ESD lesions (*p* < 0.05*).

**Conclusions:**

With the AWC, a standard endoscope can easily be transformed to double-channel functionality. We could show that EMR+ and ESD+ are non-inferior to EMR and ESD under the use of a double-channel endoscope. Consequently, the AWC presents an affordable alternative to a double-channel endoscope for both EMR and ESD.

Endoscopic mucosal resection (EMR) offers a safe, cost-effective, and well-established interventional endoscopic technique for the successful resection of many precancerous gastrointestinal lesions [1]. With its relatively low technical complexity, EMR features brief procedure times with a low risk of adverse events [2]. However, in larger lesions EMR shows a decreasing rate of en bloc resections [1, 3]. In particular, en bloc resection of laterally spreading adenomas or large sessile colorectal polyps ≥ 2 cm can be achieved in only about 30% [2, 4]. Of course, EMR can be performed in piecemeal technique. But this comes along with higher rates of incomplete resections leading to more cases of recurrence. Consequently, piecemeal resections also require more frequent follow-up endoscopies [5]. The spectrum of EMR can be extended by an external additional working channel (AWC, Ovesco Endoscopy, Tübingen, Germany). Its feasibility could already be shown in previous publications and the technique was termed “EMR+” [6–9]. Meanwhile, there have also been several reports and some studies published on the application of EMR+ in humans [7, 10, 11]. Experimental data systematically evaluating the AWC in EMR were provided by our group and others [6, 8, 9]. EMR+ with the AWC extends the range of en bloc resections beyond the relevant size of 2 cm, particularly with promising results in 3 cm lesions [6]. However, also EMR+ reaches its limits in 4 cm lesions with lower en bloc resection rates and a rising risk of perforations [6].

Consequently, endoscopic submucosal dissection (ESD) needs to be considered in lesions ≥ 3 cm. ESD has become a standard interventional endoscopic procedure also in western expert centers since its initial development in Japan [12–16]. In terms of the resection of flat lesions ≥ 3 cm, ESD offers an anatomically convincing method. Principally, with ESD en bloc resections regardless of lesion’s size can be achieved [12]. However, even for experienced endoscopists, ESD is technically challenging and associated with relevant adverse events, particularly a higher rate of perforations [2]. Moreover, it demands higher costs and more time resources. For the above reasons, also the optimization of ESD by additional endoscopic devices is needed. Many concepts of advancing ESD, e.g., by counter-traction devices, have been pursued. Taking up those approaches, also ESD can be enhanced by the AWC mounted on a standard endoscope [17]. This enables a simultaneous use of two instruments and thus the interaction of ESD knife with a grasper, ESD coagulation dissector, or other additional instruments. This technique is termed “ESD+” in analogy to EMR+ [17]. Recently, ESD+ was systematically evaluated in an animal model. Compared to conventional ESD, it could be shown that by its grasp-and-mobilizing technique, ESD+ allows potentially safer and faster resections of flat lesions [17].

In endoscopic expert centers, ESD is frequently performed with double-channel endoscopes. This offers a grasp and snare technique comparable to ESD+ [18, 19]. However, double-channel endoscopes are expensive and not cost-effective for many endoscopy centers. Consequently, in many endoscopy units, double-channel endoscopes are not available. Compared to the investment of a double-channel endoscope, additional endoscopic tools like the AWC are less expensive, and besides, they are easy to handle.

In a pre-clinical porcine ex vivo animal model, we prospectively compare the novel techniques EMR+ and ESD+ with EMR under the use of a double-channel endoscope and, respectively, ESD via a double-channel endoscope. This is carried out in order to investigate whether EMR+ and ESD+ are non-inferior to EMR and ESD via a double-channel endoscope.

## Materials and methods

This trial was a prospectively designed ex vivo study. Since no living animals or humans were included, it was exempted from IRB. The experiments were conducted at the Laboratory for Experimental Endoscopy in the Department of Gastroenterology, Gastrointestinal Oncology and Endocrinology of the University Medical Center Göttingen in Germany.

The cleaned porcine stomachs used for the experiments were defrosted prior to intervention. Afterward, they were placed into the EASIE-R simulator (Erlangen Active Simulator for Interventional Endoscopy, Endosim, LLC, Hudson, MA, USA), a well-established model for interventional endoscopic training and research that has also been evaluated at our research unit for several endoscopic procedures [6, 8, 17, 20, 21].

A well-trained senior endoscopist with previous EMR and ESD expertise in humans as well as in animal models performed all interventions (EMR+ and ESD+ with the AWC as well as EMR and ESD with the double-channel endoscope). The endoscopist was assisted by an experienced endoscopic nurse.

### Preparation of the porcine stomachs

EMR becomes particularly challenging beyond a lesion size of 2 cm [6]. Therefore, standardized flat lesions were defined in the porcine stomachs prior to intervention, measuring 3 cm or 4 cm. This was done by thermal marking with coagulation dots around a defined template. Then, the stomachs were closed by surgical suture and transferred into the EASIE-R model with esophagus and stomach fixed to the model’s plastic shell [6, 21].

### Additional working channel (AWC)

In analogy to the setup known from the full-thickness resection device (FTRD), the AWC can be mounted at the tip of a standard endoscope. The AWC features a shaft with a length of either 122 cm (endoscope insertion length: 103–110 cm) or 185 cm (endoscope insertion length: 160–170 cm). It has a flexible attachment for endoscope diameters from 8.5 to 13.5 mm. Via an adaptor, the AWC is fixed at the endoscope handle. A valve can be connected to the adaptor via Luer-lock. The AWC comes along with a sleeve and an adhesion tape.

Instruments with an outer diameter of up to 2.8 mm can be introduced via the AWC [6].

**Fig. 1:**
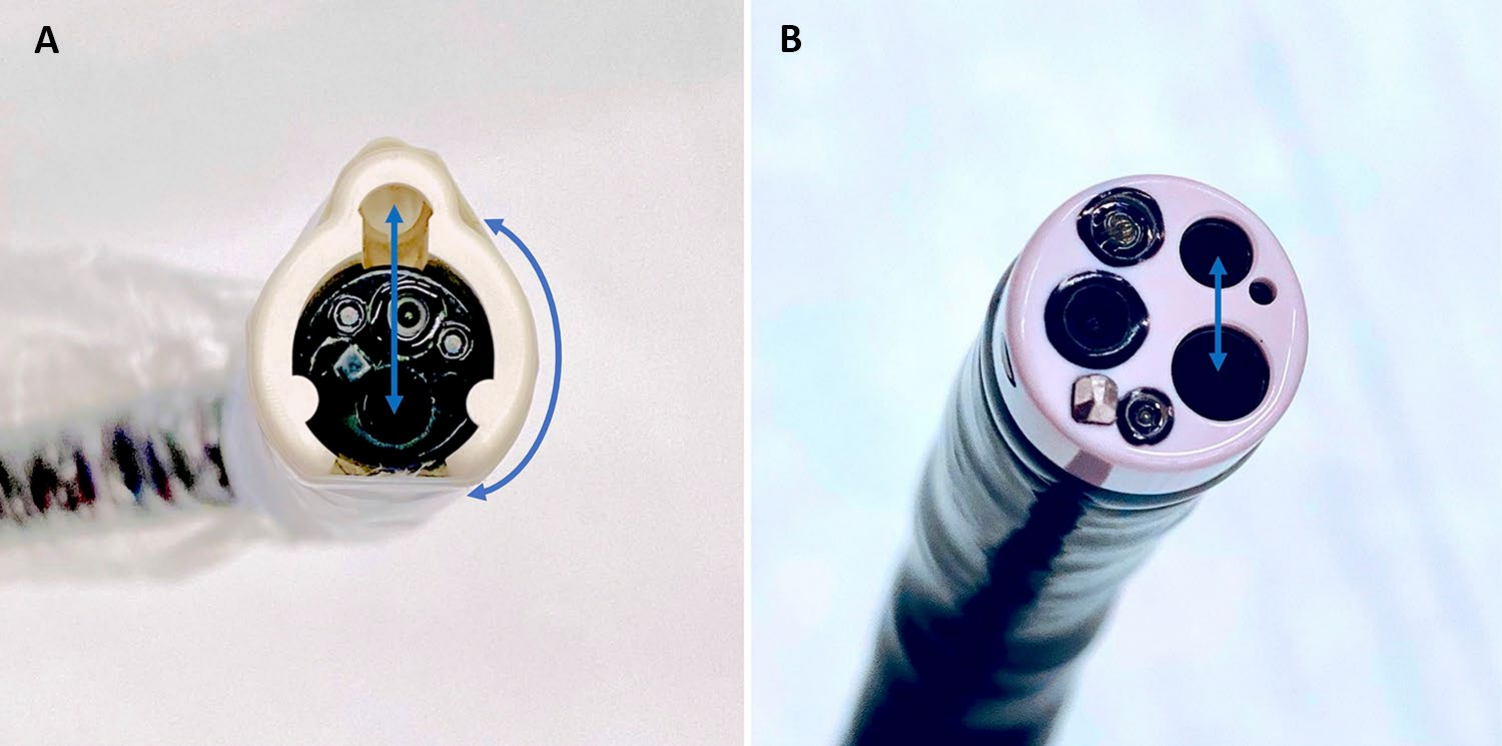
**A** Frontal view of the AWC mounted on a standard gastroscope with a wide distance (approximately 8 mm) between the working channels (straight arrow) and the choice of flexible positioning (curved arrow). **B** Frontal view of a double-channel endoscope with the working channels in narrow and fixed distance (arrow)

**Fig. 2:**
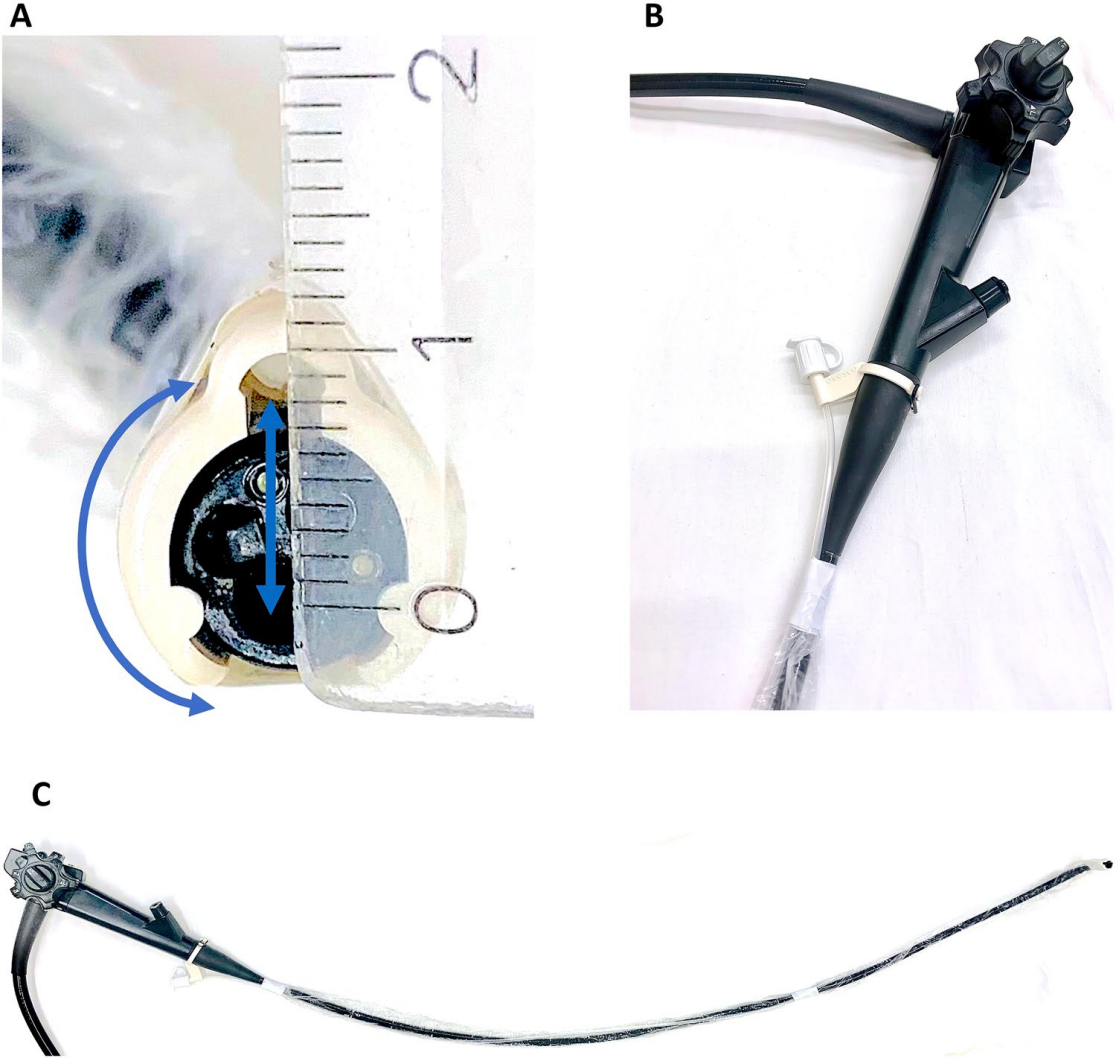
**A** AWC mounted on the tip of a standard gastroscope with the distance to the regular working channel (approximately 8 mm, depending on the particular endoscope). **B** AWC valve attached to the shaft of the endoscope. **C** External installation of the AWC on a single-channel gastroscope

Principally, the AWC can be rotated 360° on the distal tip of the endoscope. In our experiments, all AWC procedures were conducted with the AWC in the counterpart position to the working channel (Fig. 1A). The AWC setup is also illustrated in Fig. 2.

### Procedure of EMR+ and ESD+

**Fig. 3:**
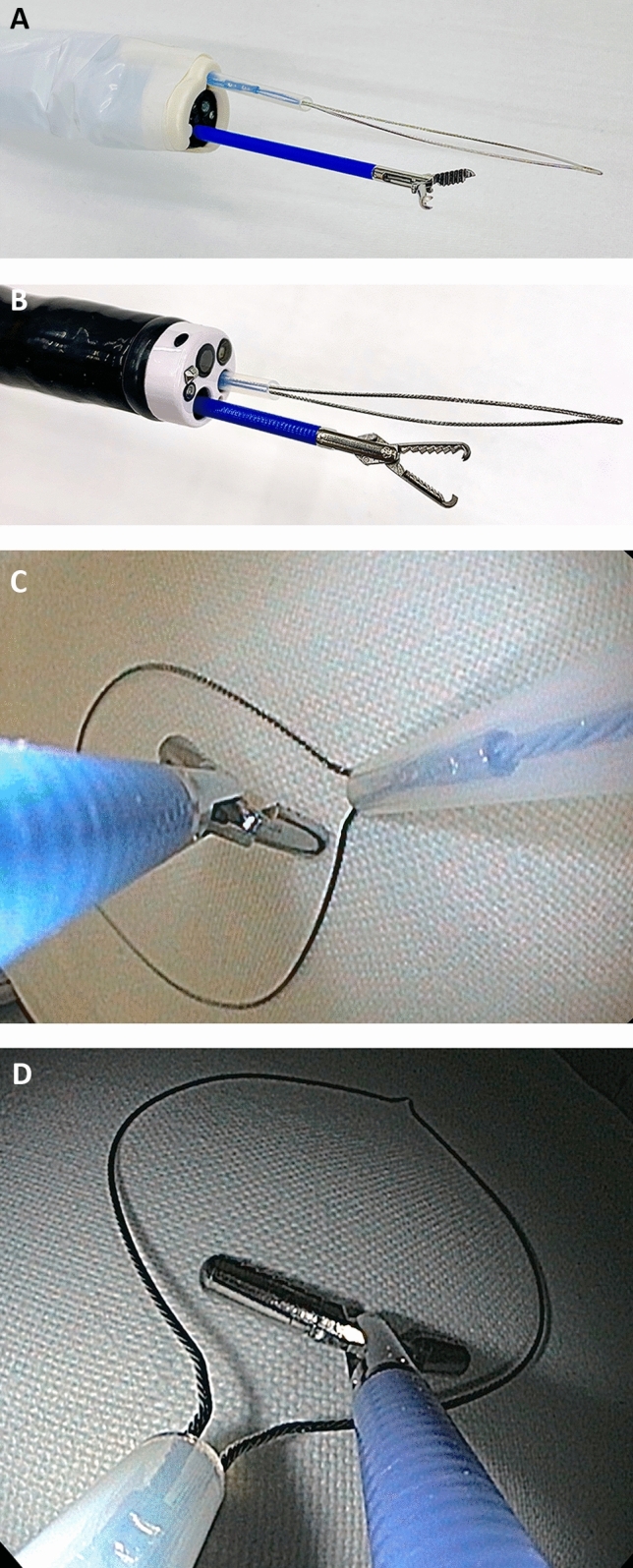
**A** AWC setup for EMR+ with grasper and snare. **B** Double-scope setup for EMR DC with grasper and snare. **C** Endoscopic view: EMR+ with grasper and snare. **D** Endoscopic view: EMR DC with grasper and snare

**Fig. 4:**
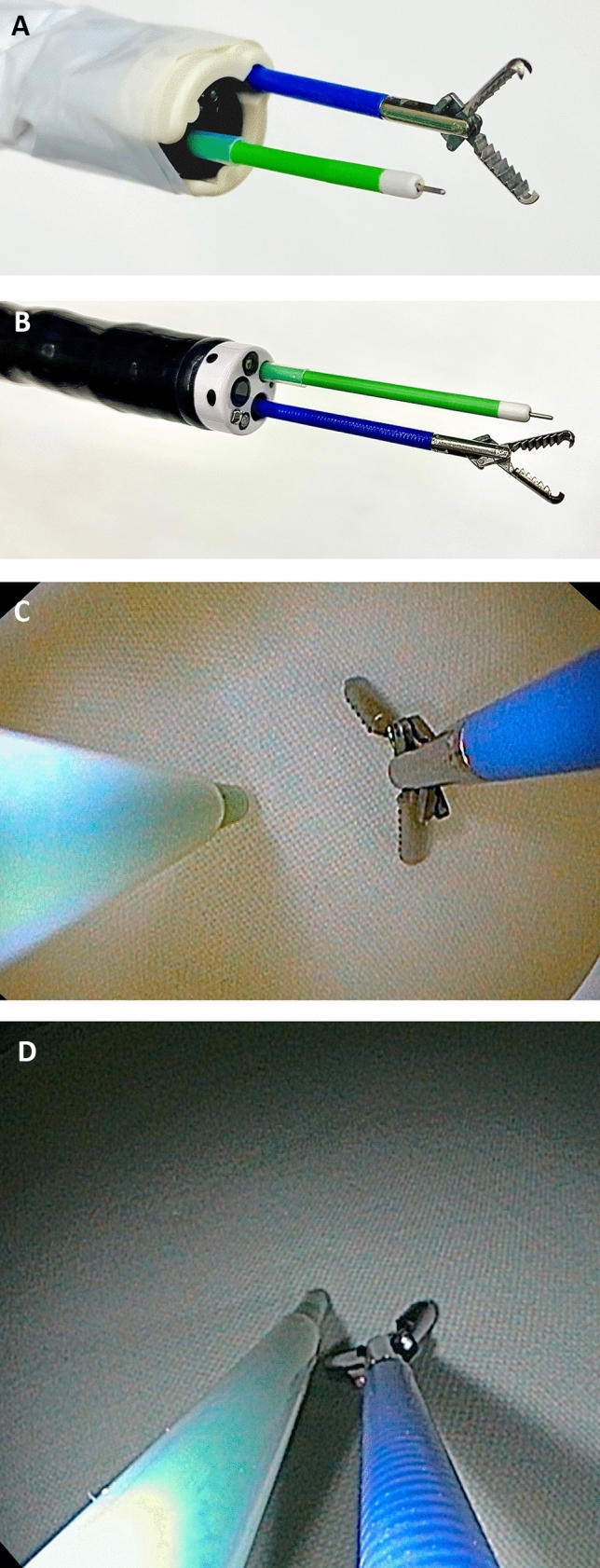
**A** AWC setup for ESD+ with grasper and AqaNife 2.5. **B** Double-scope setup for ESD DC with grasper and AqaNife 2.5. **C** Endoscopic view: ESD+ with grasper and AqaNife 2.5. **D** Endoscopic view: ESD DC with grasper and AqaNife 2.5

**Fig. 5:**
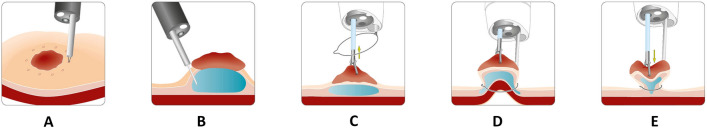
Principle of EMR+ procedure. **A** Target lesion. **B** Submucosal injection. **C** Positioning of snare and grasper. **D** Elevation of the lesion and snare closure. **E** Pushback of the grasper while snare stays closed followed by resection (*Source* with permission from Ovesco Endoscopy AG, Tübingen, Germany)

**Fig. 6:**
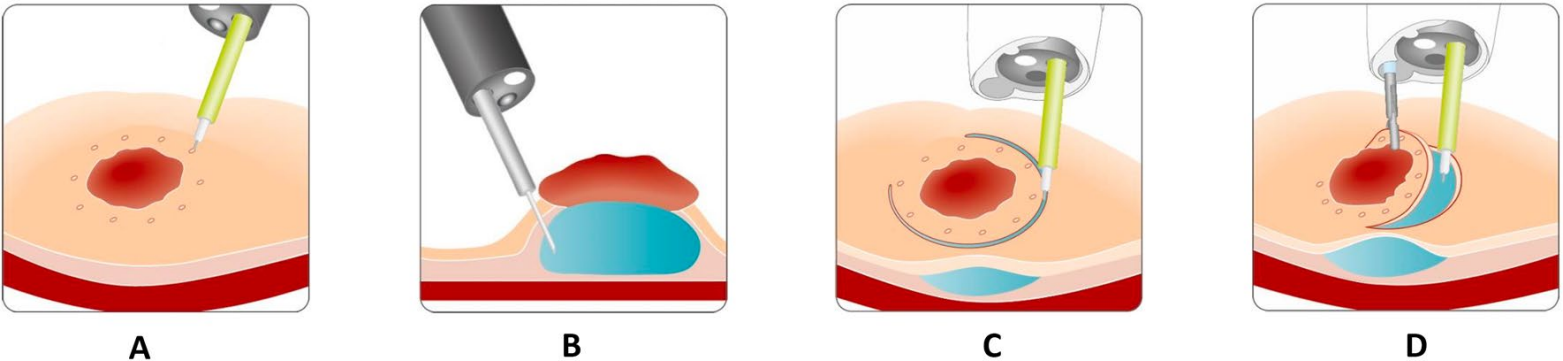
Principle of ESD+ procedure. **A** Marking of the target lesion. **B** Submucosal injection. **C** Circumferential ESD incision. **D** Mobilization of the lesion’s flap with a grasper introduced via the AWC (*Source* with permission from Ovesco Endoscopy AG, Tübingen, Germany)

EMR+ and ESD+ were conducted with a conventional gastroscope (EG-530WR Fujinon, Fujifilm, Tokyo, Japan), the AWC device (Ovesco Endoscopy, Tübingen, Germany) and, in the case of ESD+, with the AqaNife 2.5 mm needle length. Setup and principle of EMR+ and ESD+ technique with the help of the AWC are shown in Figs. 3, 4, 5, and 6.

### Procedure of EMR and ESD with the double-channel endoscope

EMR and ESD were performed with the double-channel endoscope EG-530D Fujinon, Fujifilm, Tokyo, Japan and, in the case of ESD, with the AqaNife 2.5 mm needle length. The setup of EMR DC and ESD DC is shown in Figs. 3 and 4.

In all resections, the FTRD grasper (Ovesco Endoscopy, Tübingen, Germany) was used. In EMR and EMR+ resections, a 33-mm snare (Boston Scientific, Marlborough, Massachusetts, United States) was applied. In both ESD techniques, the ESD injection fluid was Hydroxyethyl starch (HAES) mixed with methylene blue dye for better visualization and optimal tissue differentiation. The electrosurgical unit was ERBE VIO 200 D (ERBE Elektromedizin, Tübingen, Germany) with the mode EndoCut Q.

### Data collection

By an independent observer, the following parameters were recorded:

- *Primary end point* Rate of en bloc resection.
- *Secondary end points* Time of procedure for EMR+ and ESD+ as well as for EMR and ESD via double-channel endoscope (minutes), adverse events (muscularis damage, perforations).

After every resection the specimens were spread out and pinned on cork plates. The en bloc resection was evaluated and documented. En bloc resection was defined as the complete resection with all previously marked coagulation dots within the resected specimen. All procedures were intended to be en bloc resections. The procedure time was defined from submucosal injection of the lesion until its complete resection. Every resection site was visually inspected for muscular damage. By an insufflation test of the porcine stomach, potential perforations were evaluated.

### Statistical analysis

Data analysis was performed with SPSS Version 28.0.1.1 (IBM, Armonk, NY, USA) and Prism 9 for macOS Version 9.4.1 (GraphPad Software, LLC, San Diego, CA, USA). The analysis of adverse events and en bloc resection rates was conducted with Fisher’s Exact Test. The time of procedure was analyzed by Mann–Whitney *U* Test. As usual, we considered *p* values less than 0.05 as statistically significant. They are marked by asterisk.

## Results

**Fig. 7:**
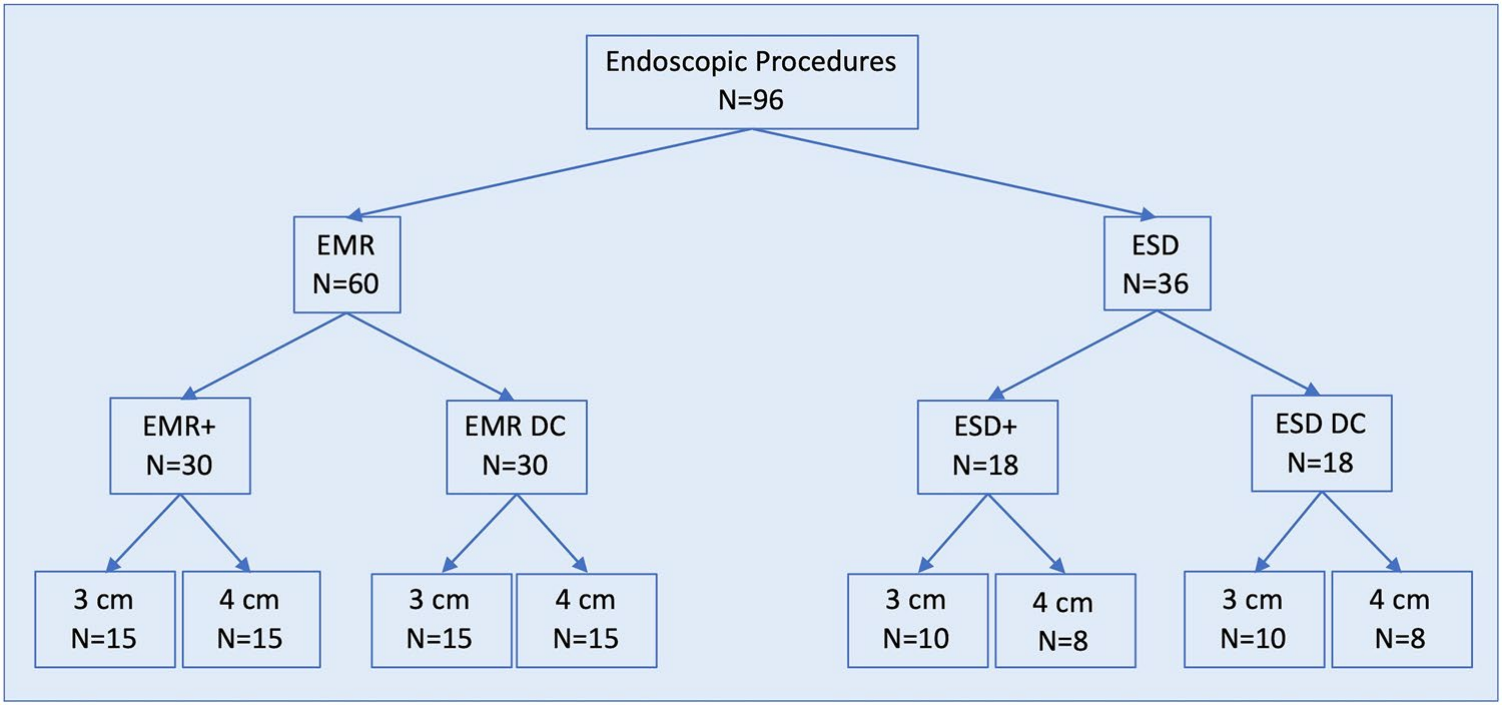
Study design

Lesions with two different sizes with a diameter of 3 cm and 4 cm were set in the EMR as well as in the ESD groups (Fig. 7). Altogether, we used 11 porcine stomachs, each with 6–9 lesions, dependent on the lesions’ and stomachs’ sizes.

Overall, 96 endoscopic procedures were conducted in the porcine ex vivo model (Fig. 7). In detail, we performed 15 EMR+ vs. 15 EMR with the double-channel endoscope (DC) in 3 cm lesions and also 15 EMR+ vs. 15 EMR DC in 4 cm lesions. We conducted 10 ESD+ vs. 10 ESD DC in 3 cm lesions and 8 ESD+ vs. 8 ESD DC in 4 cm lesions (Fig. 7).

### Primary end point

#### Rate of en bloc resection

In 3 cm lesions, EMR+ reached an en bloc resection rate of 73.33% (11/15) compared to 60.00% (9/15) with EMR DC, *p* = 0.70.

In 4 cm lesions, EMR+ reached an en bloc resection rate of 60.00% (9/15) compared to 46.67% (7/15) with EMR DC, *p* = 0.72.

Both ESD+ and ESD DC showed an en bloc resection rate of 100% in all lesions’ sizes (36/36).

### Secondary end points

#### Time of procedure dependent on size

In all groups, the mean procedure time was shorter in 3 cm lesions compared to 4 cm lesions [EMR+ 6.07 (SD 2.28) vs. 8.13 (SD 2.97) min, *p* = 0.48; EMR DC 7.13 (SD 2.23) vs. 9.20 (SD 2.46) min, *p* = 0.02*; ESD+ 21.60 (SD 5.17) vs. 29.25 (SD 7.36) min, *p* = 0.03*; ESD DC 26.60 (SD 5.19) vs. 35.75 (SD 6.27) min, *p* < 0.01*].

#### Time of procedure dependent on technique

##### EMR+ vs EMR DC in 3 cm lesions

**Fig. 8:**
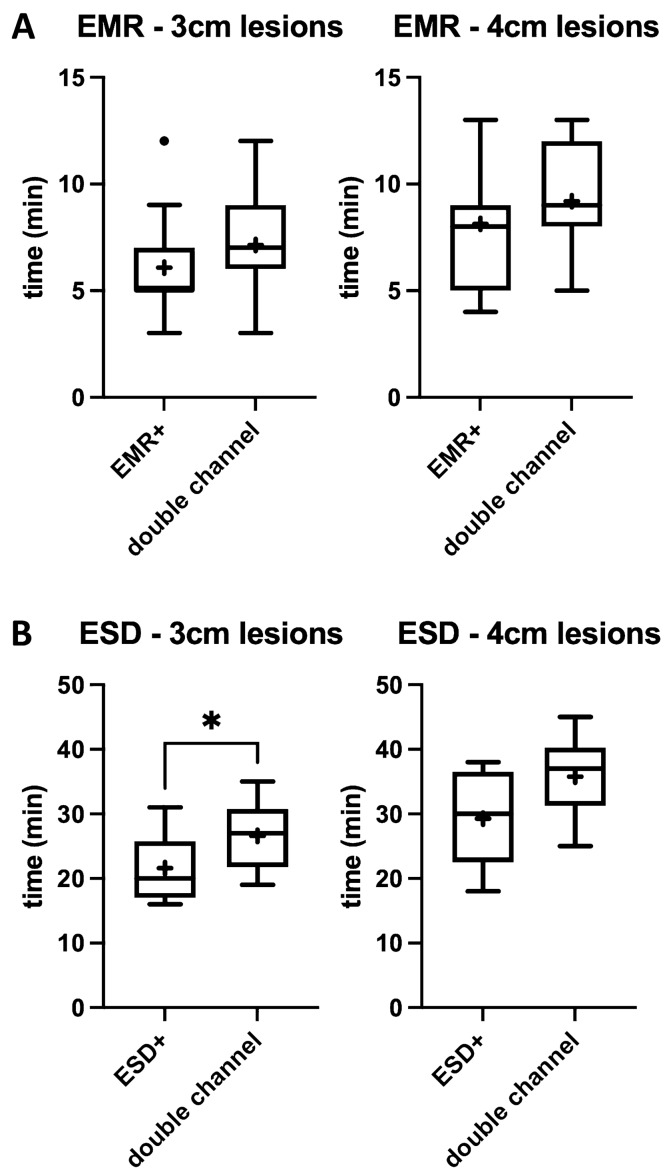
**A** Time of procedure, EMR groups. **B** Time of procedure, ESD groups

In 3 cm lesions, EMR+ was faster than EMR DC although without statistical significance [6.07 min (SD 2.28) vs. 7.13 min (SD 2.23), *p* = 0.12] (Fig. 8A).

##### EMR+ vs EMR DC in 4 cm lesions

Also, in 4 cm lesions, EMR+ was faster than EMR DC although without statistical significance [8.13 min (SD 2.97) vs. 9.20 min (SD 2.46), *p* = 0.28] (Fig. 8A).

##### ESD+ vs ESD DC in 3 cm lesions

In 3 cm lesions, ESD+ was significantly faster than ESD DC [21.60 min (SD 5.17) vs. 26.60 min (SD 5.19), *p* < 0.05*] (Fig. 8B).

##### ESD+ vs ESD DC in 4 cm lesions

In 4 cm lesions, ESD+ was faster than ESD DC although without statistical significance [29.25 min (SD 7.36) vs. 35.75 min (SD 6.27), *p* = 0.07] (Fig. 8B).

#### Safety and adverse events

In 3 cm lesions, no perforations or muscularis damages occurred neither in the EMR nor in the ESD groups.

In 4 cm lesions, we observed 3 (3/15, 20.00%) muscularis damages with EMR+ and 2 (2/15, 13.33%) muscularis damages with EMR DC (*p* ≥ 0.99). Also, in 4 cm lesions, there was 1 (1/8, 12.50%) muscularis damage under ESD+ as well as 1 (1/8, 12.50%) under ESD DC (*p* ≥ 0.99).

Throughout the whole study, no perforations with an air leakage in the insufflation tests occurred.

## Discussion

EMR+ and ESD+ combine EMR and ESD with the grasp technique of the recently launched AWC. In this study, we aimed to evaluate EMR+ and ESD+ compared to EMR and ESD under the use of a double-channel endoscope in a porcine ex vivo model. To the best of our knowledge, this was conducted systematically for the first time.

Over time, the evolution of endoscopic resection techniques has continuously moved forward from EMR to ESD or e.g., endoscopic full-thickness resection (EFTR) with the full-thickness resection device (FTRD) in particular indications [22–24]. A lot of research effort has been undertaken in order to secure and accelerate endoscopic resections. In particular, the endeavor of achieving better intraluminal tissue traction has been a central focus of endoscopic research. For this purpose, a variety of additional endoscopic devices has been designed such as rubber bands, external forceps, clips with attached strings, magnetic anchors, or a pulley system with clips to facilitate endoscopic traction [25–32]. So far, the optimal traction device for endoluminal surgery has still not been found. That is why we need ongoing basic research on this aim.

Compared to EMR, ESD is a reliable and elegant technique for extended endoscopic resections featuring higher rates of R0 resections and consequently lower rates of recurrence [33]. With regard to the treatment of early gastric cancer, several meta-analyses compared ESD with EMR showing more en bloc resections, higher histologically complete resection rates, and lower recurrence frequencies for ESD [12, 34–36]. However, the superiority of ESD applies only for its use in the hand of an ESD-experienced endoscopic expert since ESD is complex and technically challenging. Therefore, ESD features a flat learning curve also for well-trained endoscopists and is associated with a relevant rate of adverse events, particularly perforations in up to 4–10% [12, 37].

To address these challenges, ESD+ was developed in order to improve feasibility and safety of ESD. Similar to EMR+, its principle is based on the AWC [8, 10, 17].

Certainly, in EMR as well as in ESD, a double-channel endoscope can be used to achieve better tissue traction with a simultaneous grasp and snare technique [18, 19, 38–40]. However, in many endoscopy units a double-channel endoscope is not available as it is an expensive investment. Its fixed and narrow distance between the two working channels of a double-channel endoscope and the fact that they are aligned in parallel, results in a lack of overview, flexibility, and sufficient triangulation [6, 17] (Figs. 1B, 3B, D, 4B, D). Compared with this, the AWC is more distant to the endoscope’s working channel and proceeds in a pointed angle. Also more variable positions of the AWC in relation to the endoscope’s working channel can be achieved by turning the AWC’s cap to the required position [7] (Figs. 1A, 2A). Altogether, this can lead to a more flexible triangulation of the endoscopic instruments consequently resulting in a better visibility and improved bimanual endoscopic working [6, 17]. The AWC might come along with certain disadvantages. Due to its external fixation outside the endoscope, the entire diameter of the scope tip increases which can possibly make the passage more difficult. Also, with the mounted AWC the endoscope becomes more rigid and maneuvers could become less comfortable to undertake. Although we did not encounter relevant drawbacks in our model these points could become relevant in clinical practice.

In our study, EMR+ and EMR DC provide convincing data in terms of en bloc resection rates and safety in 3 cm lesions but both come to technical limits in 4 cm lesions. This validates the previous data [6]. We can demonstrate that ESD is reliable concerning en bloc resection rates (100% in all groups) and safety, although there is a little increase of adverse events (muscularis damage) in 4 cm lesions with ESD+ as well as with ESD DC (both from 0.00 to 12.50%). This also confirms the previous data [17]. As well known from daily clinical routine, procedure time rises with size of the lesion. This can be recapitulated by our results in all applied techniques (EMR+, EMR DC, ESD+, and ESD DC). In our trial, all study arms with the AWC (EMR+ and ESD+) show shorter resection times compared to the study arms under use of the double-channel endoscope although this only reaches significance in 3 cm ESD lesions. However, our study fulfills its aim as it shows a non-inferiority of both EMR+ and ESD+ with the AWC compared to EMR and ESD under the use of a double-channel endoscope.

Our prospective study was conducted in a well-established porcine ex vivo model. This comes along with inherent limitations concerning transferability to living humans. The model can obviously not recapitulate bleeding, tissue movement, and other physiological features, e.g., neoplastic recurrence and stricture outcome. Also, a histopathological examination is not expedient. Furthermore, pigs have a thicker gastric mucosa and consequently a higher mucosal rigidity compared to humans. This may affect technical opportunities of all techniques applied in our study. Due to our experimental setup, in all groups we sought for a homogenous arrangement of the lesions’ positions (antegrade vs. retrograde). Since the study design would have become too confusing, we explicitly decided not to further subdivide our study arms to different positions of the lesions. Therefore, this study is not randomized which can also be regarded as a limitation.

## Conclusion

EMR+ and ESD+ under use of the AWC allow fast and safe endoscopic resections. With the AWC, a standard single-channel endoscope can easily be transformed to double-channel functionality leading to better intraluminal tissue control.

In the ex vivo porcine model, we could show that EMR+ and ESD+ are not less than equivalent to EMR and, respectively, ESD under the use of a double-channel endoscope. As double-channel endoscopes are expensive investments for endoscopy units, the AWC presents an affordable alternative with good applicability in endoscopic everyday practice as well in the case of EMR+ as with ESD+.

## Abbreviations

AWC: Additional working channel
EFTR: Endoscopic full-thickness resection
EMR: Endoscopic mucosal resection
EMR+: Endoscopic mucosal resection using the AWC
EMR DC: Endoscopic mucosal resection using a double-channel endoscope
ESD: Endoscopic submucosal dissection
ESD+: Endoscopic submucosal dissection using the AWC
ESD DC: Endoscopic submucosal dissection using a double-channel endoscope
FTRD: Full-thickness resection device
HAES: Hydroxyethyl starch
IRB: Institutional Review Board (Ethikkommission)
min: Minute
R0: No residual tumor
SD: Standard deviation
